# In-depth profiling and analysis of host and viral microRNAs in Japanese flounder (*Paralichthys olivaceus*) infected with megalocytivirus reveal involvement of microRNAs in host-virus interaction in teleost fish

**DOI:** 10.1186/1471-2164-15-878

**Published:** 2014-10-08

**Authors:** Bao-cun Zhang, Jian Zhang, Li Sun

**Affiliations:** Key Laboratory of Experimental Marine Biology, Institute of Oceanology, Chinese Academy of Sciences, Qingdao, 266071 China; Graduate University of the Chinese Academy of Sciences, Beijing, 100049 China; Collaborative Innovation Center of Deep Sea Biology, College of Life Sciences, Zhejiang University, Hangzhou, 310058 China

**Keywords:** Iridovirus, microRNA, *Paralichthys olivaceus*, Teleost, Virus-host interaction

## Abstract

**Background:**

MicroRNAs (miRNAs) regulate gene expression by binding to mRNA transcripts in various biological processes. In mammals and birds, miRNAs are known to play vital parts in both host immune defense and viral infection. However, in lower vertebrates such as teleost, systematic investigations on host and viral miRNAs are lacking.

**Results:**

In this study, we applied high-throughput sequencing technology to identify and analyze both host and viral miRNAs in Japanese flounder (*Paralichthys olivaceus*), an economically important teleost fish farmed widely in the world, infected with megalocytivirus at a timescale of 14 days divided into five different time points. The results showed that a total of 381 host miRNAs and 9 viral miRNAs were identified, the latter being all novel miRNAs that have no homologues in the currently available databases. Of the host miRNAs, 251 have been reported previously in flounder and other species, and 130 were discovered for the first time. The expression levels of 121 host miRNAs were significantly altered at 2 d to 14 d post-viral infection (pi), and these miRNAs were therefore classified as differentially expressed host miRNAs. The expression levels of all 9 viral miRNAs increased from 0 d pi to 10 d pi and then dropped from 10 d pi to 14 d pi. For the 121 differentially expressed host miRNAs and the 9 viral miRNAs, 243 and 48 putative target genes, respectively, were predicted in flounder. GO and KEGG enrichment analysis revealed that the putative target genes of both host and viral miRNAs were grouped mainly into the categories of immune response, signal transduction, and apoptotic process.

**Conclusions:**

The results of our study provide the first evidences that indicate existence in teleost fish (i) infection-responsive host and viral miRNAs that exhibit dynamic changes in expression profiles during the course of viral infection, and (ii) potential involvement of miRNAs in host-viral interaction.

**Electronic supplementary material:**

The online version of this article (doi:10.1186/1471-2164-15-878) contains supplementary material, which is available to authorized users.

## Background

MicroRNAs (miRNAs) are small non-coding RNAs with ~22 nucleotides (nt) in length. They have been recognized as crucial regulators of gene expression in plants and animals through interaction with specific mRNA targets and thereby affecting translation [1]. Functionally, miRNAs targeting on gene expression are involved in a range of biological processes, such as development, immunity, and apoptosis [2]. Dysregulation of miRNA production is often associated with diseased conditions, e.g. aberrant miRNA expression is a characteristic of all types of cancer. Consequently, miRNAs have been used as diagnostic and prognostic markers for cancer and other diseases [1, 3–7].

MicroRNAs are key players in the regulation of various host-pathogen interactions. Accumulating reports have indicated that virus can produce miRNAs to manipulate host gene expression and facilitate viral replication [8]. On the other hand, the host limits viral infection through differentially expressing the miRNAs that target essential viral genes [9–12]. Compared to viral proteins, viral miRNAs are non-immunogenic, require less coding capacity, and can evolve rapidly to target new transcripts. Since the discovery of the first viral miRNAs in human B cells infected with Epstein-Barr virus (EBV) [13], more than 200 viral miRNAs have been identified in herpes virus, polyomavirus, ascovirus, and adenovirus [14]. Viral miRNAs contribute to infection through various mechanisms, notably immune evasion [15, 16], induction of anti-apoptosis [17], reducing the antigenicity of viral proteins [16, 18], and promoting cell survival, proliferation, and differentiation [14].

With the development of high-throughput sequencing technology, more and more miRNAs associated with host-virus interactions have been discovered. These interactions include those between cytomegalovirus and humans [19], H1N1 influenza virus and mouse [20], avian influenza virus and chicken [21], and, for aquaculture virus, white spot syndrome virus (WSSV) and shrimp [22]. In lower vertebrates, a few studies on miRNAs associated with bacterial infection have been reported [23–25], however, systematic investigations on the role of miRNAs in interactions between lower vertebrate hosts (such as teleost) and viruses have not yet been documented.

*Iridoviridae* is a family of large viruses with linear, double-stranded DNA. The family is subdivided into five genera, of which, three genera, i.e., *Lymphocystivirus*, *Ranavirus*, and *Megalocytivirus,* are serious pathogens to a wide range of freshwater and marine fish including rock bream, turbot, Japanese flounder, orange-spotted grouper, catfish, sturgeon, largemouth bass, rainbow trout, and red sea bream [26–30]. However, the infection mechanism of iridovirus is largely unknown.

In this study, using Japanese flounder, an important economic teleost species, as a host model, we examined viral and host miRNAs associated with megalocytivirus infection by the approach of high-throughput sequencing. In addition, to facilitate understanding of the functional attributes of the miRNAs, the target genes of viral miRNAs and differentially expressed host miRNAs were predicted by in silico analysis and the gene ontology categories of the putative targets are described. Our results indicate for the first time that in teleost fish, the expression profiles of both host and viral miRNAs changed dynamically during the course of viral infection, and that miRNAs are most likely involved in the process of host-viral interaction.

## Methods

### Animal ethics

Experiments involving live animals were conducted in accordance with the "Regulations for the Administration of Affairs Concerning Experimental Animals" promulgated by the State Science and Technology Commission of Shandong Province. The study was approved by the Ethics Committee of Institute of Oceanology, Chinese Academy of Sciences.

### Fish

Clinically healthy juvenile Japanese flounder (average size 11.3 cm; average weight 12.4 g) were purchased from a commercial fish farm in Shandong Province, China and maintained at ~22°C in aerated seawater. Fish were acclimatized in the laboratory for two weeks before experimental manipulation. Before experiment, fish were randomly sampled for the examination of bacteria and megalocytivirus in blood, liver, kidney, and spleen as reported previously [31, 32]. No bacteria or virus were detected from the examined fish. Before tissue collection, fish were euthanized with an overdose of tricaine methanesulfonate (Sigma, St. Louis, MO, USA) as reported previously [33].

### Experimental infection

The fish viral pathogen megalocytivirus RBIV-C1 [32] was suspended in PBS to 1 × 10^6^ copies/ml. Japanese flounder (~12.4 g) were divided randomly into two groups and injected intraperitoneally with 100 μl viral suspension. At 0 day (d), 2 d, 6 d, 10 d, and 14 d post-infection (pi), fish (three at each time point) were euthanized, and spleen was collected under aseptic conditions and immediately stored in liquid nitrogen for later use. At each time point the spleen tissues of three fish were pooled together and used for subsequent small RNA sequencing.

### Sequencing of small RNAs

Small RNA isolation, library construction, and high-throughput sequencing were all carried out by LC Science, Houston, USA. Briefly, total RNA was isolated from spleen tissues using *mir*Vana™ miRNA Isolation Kit (Life Technologies, USA) according to the manufacturer’s instructions. The quantity and purity of the RNA were monitored using a NanoDrop™ 1000 spectrophotometer (Thermo Fisher Scientific, WI, USA) at *A*_260_/*A*_280_ > 2.0. The integrity of the RNA was analyzed using the Agilent 2100 Bioanalyzer system and RNA 6000 Nano LabChip Kit (Agilent, CA, USA) with RNA integrity number (RIN) > 8.0. Approximately 1 μg of total RNA was used to prepare small RNA library (16–30 nt) with TruSeq Small RNA Sample Prep Kits (Illumina, San Diego, USA), and single-end sequencing (36 bp) was performed on an Illumina Hiseq2000 by LC Science (Houston, USA).

### Analysis of sequencing reads

A proprietary pipeline script, ACGT101-miR v4.2 (LC Sciences), was used for following sequencing data analysis: (i) generating unique families of sequenced sequences (SequSeqs) by sorting raw sequencing reads; (ii) filtering SequSeqs, in which the impurity sequences due to sample preparation, sequencing chemistry and processes, and the optical digital resolution of the sequencer detector were removed; (iii) discarding SequSeqs mapped to RFam database (http://rfam.janelia.org) and RepBase (http://www.girinst.org/repbase); (iv) mapping miRNA Unique Seqs to pre-miRNA (mir) and expressed sequence tag (EST), during which various mappings were performed on unique sequences against mir and mature miRNA (miR) sequences listed in miRBase 20.0 (http://www.mirbase.org/search.shtml) with an E value similarity cutoff of 1e-10. Meanwhile, Bowtie-1.0.0 (http://bowtie-bio.sourceforge.net) was also applied to find the reads mapped to the miRbase with the following criteria: (i) the length of seed sequence is 16 nt, and (ii) the error allowable in seed region is 1 nt. To identify novel miRNA, the remaining high-quality sequences that have no homologues in miRBase 20.0 but can map to the hairpin structures of flounder ESTs or RBIV-C1 genome were further analyzed and considered candidates of novel miRNA if they possess the following characteristics: (i) number of nucleotide in one bulge in stem < = 12, (ii) number of base pair in the stem region of the predicted hairpin > =16, (iii) cutoff of free energy (kCal/mol) < = -15, (iv) length of hairpin (up and down stems + terminal loop) > =50, (v) length of hairpin loop < =20, (vi) number of nucleotide in one bulge in mature region < = 4, (vii) number of biased error in one bulge in mature region < =2, (viii) number of biased bulge in mature region < =2, (ix) number of error in mature region < = 4, (x) number of base pair in the mature region of the predicted hairpin > =12, (xi) percent of mature region in stem > =80 [34]. For cross-comparisons of miRNAs at different time points of infection, the number of miRNA reads was normalized with Bonferroni correction, and significant expression differences were assessed with Chi-square test and Fisher exact test.

### Real-time quantification of miRNAs by stem–loop RT-PCR

Total RNAs from the spleens of Japanese flounder at different infection times were isolated using RNAiso plus kit (TaKaRa, Dalian, China) and then treated with DNase I (TaKaRa, Dalian, China) to remove remaining DNA. One microgram of total RNA was reverse transcribed to cDNA using RevertAid First Strand cDNA Synthesis Kit (Thermo Scientific Fermentas, Beijing, China) and stem-loop reverse transcription (RT) primers (Additional file 1). The mix was incubated at 42°C for 60 min and then at 70°C for 5 min. Quantitative real-time PCR was carried out in an Eppendorf Mastercycler (Eppendorf, Hamburg, Germany) in a 20 μl reaction volume containing 1 μl cDNA, 10 μl SYBR Premix Ex Taq™II (TaKaRa, Dalian, China), 0.2 μl specific forward primer (20 μM), 0.2 μl reverse primer (20 μM), and 8.6 μl water. The reaction was performed at 95°C for 5 min, followed by 45 cycles of 94°C for 15 s, 60°C for 15 s, and 72°C for 15 s. The abundance of miRNAs was normalized relative to that of 5S rRNA as reported previously [35]. All reactions were performed in triplicate. The threshold cycle (Ct) was determined using the default threshold settings, and the data were analyzed using 2^–ΔΔCt^ program. The experiment was performed independently three times.

### Prediction and analysis of the target genes of miRNAs

The putative 3’- untranslated regions (UTRs) of flounder mRNAs were used for the prediction of miRNA target genes with TargetScan 6.2 (http://www.targetscan.org/) and miRanda (http://www.microrna.org/). TargetScan was used to search for miRNA seed matches (nucleotides 2–8 from the 5' end of miRNA) in 3'-UTR sequences. miRanda was used to match the entire miRNA sequences. The parameters of TargetScan and miRanda were set as score >50 and free energy < -20 kcal/mol respectively. The results predicted by the two algorithms were combined, and the overlaps were calculated. Enrichment analysis of the predicted target genes was conducted with Gene Ontology (GO) (http://www.geneontology.org/) and KEGG pathway (http://www.genome.jp/kegg/). A heatmap chart was drawn by transforming the normalized data to a log 2 scale for visualization purpose. Hierarchical clustering was performed using the gplots heatmap.2 of R program.

## Results

### Identification of host (Japanese flounder) miRNAs

**Figure 1:**
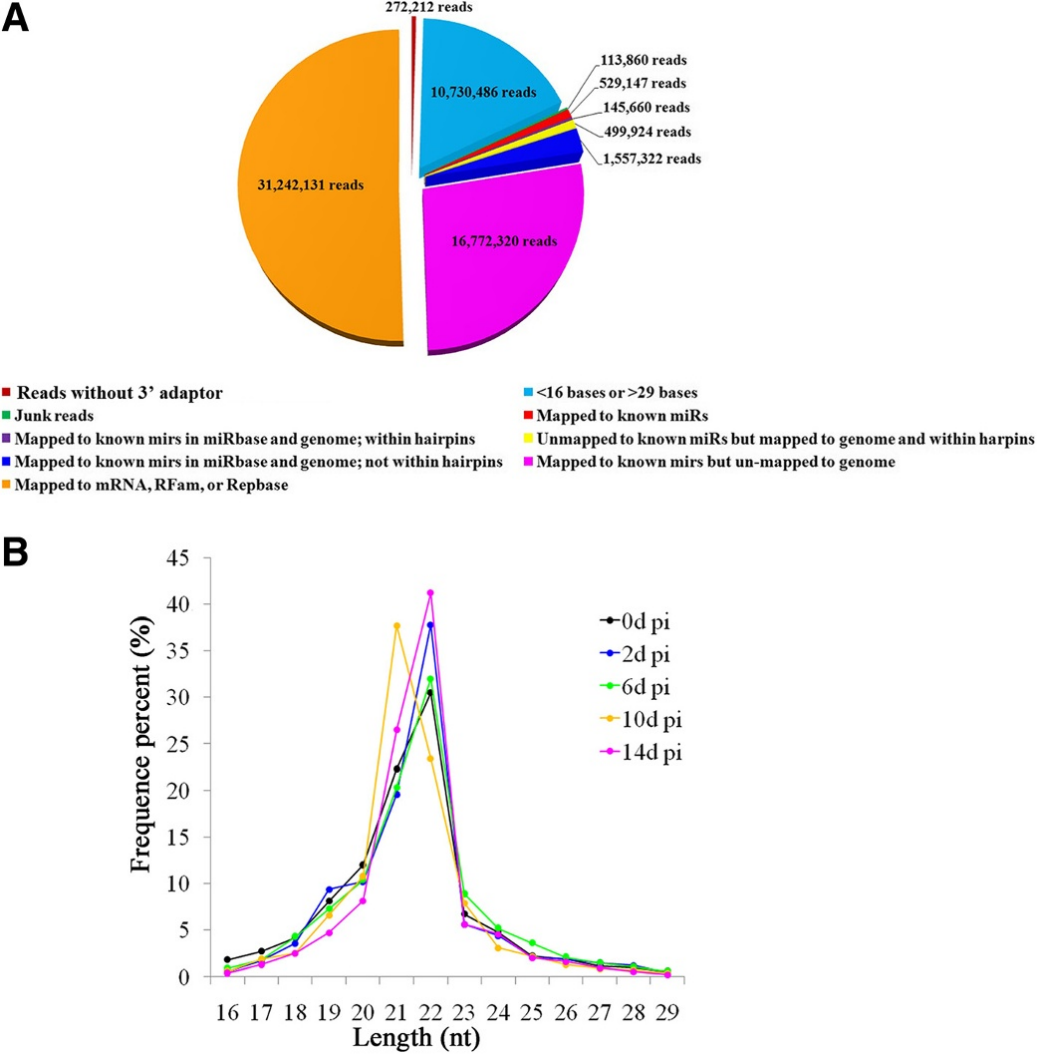
**Analysis of the sequencing reads of flounder small RNAs at different times of megalocytivirus infection. (A)** Summary of the sequencing reads. **(B)** Length distributions of the sequencing reads after removing 3’ adaptor-missing reads, <16 bases reads, >29 bases reads, junk reads, and reads mapped to RFam database and RepBase. nt, nucleotide.

In order to identify the miRNAs involved in viral infection and host immune response, Japanese flounder were infected with megalocytivirus for 0 d, 2 d, 6 d, 10 d, and 14 d. Small-fragment RNA libraries representing the five time points were constructed and subjected to sequence analysis. A total of 61,863,062 raw sequences were obtained. After removing reads without 3’ adaptor, <16-base reads, >29-base reads, and junk reads, 50,746,504 reads were mappable to RFam database, RepBase, miRBase 20.0, the hairpin regions of flounder EST database, and the hairpin regions of the genome sequence of megalocytivirus RBIV-C1 (GenBank accession no. KC244182) (Figure 1A). For the sequences mapped to miRBase 20.0 and the hairpin regions, length distribution analysis indicated that the majority of sequences were distributed in the 20–23 nucleotides (nt) range, and that 22-nt sequences were the most abundant, with the exception for the 10 d post-infection (pi) group, which contains predominantly 21-nt sequences (Figure 1B). BLAST against miRBase 20.0 showed that, with an E-value similarity cutoff of 1e–10 and allowing for one or two mismatches, 26 reads were mapped to the known miRNAs of flounder, and 225 reads show sequence similarities with the miRNAs of other bony fishes as well as reptiles, birds, and mammals (Additional file 2). Hence, 251 miRNAs with known homologues were found. For the remaining reads, 2,588 were mapped to the hairpin structures of flounder ESTs. Further analysis (based on the principles described in Methods, section “Analysis of sequencing reads”) revealed that 130 of the 2,588 reads are novel flounder miRNAs which have no known homologues (Additional file 2, pol-miR-3p-1043040_2 to pol-miR-5p-96_67419). Taken together, a total of 381 flounder miRNAs were identified.

Of the 251 flounder miRNAs that have known homologues in different animal species, pol-miR-133a-3p_L-1R + 1 is most widely distributed, with counterparts existing in zebrafish, Japanese killifish, mouse, humans, common chimpanzee, pig, platypus, Carolina anole, pipid frog, chicken, and zebra finch (Additional file 2). Other flounder miRNAs with relatively abundant distributions in vertebrates and invertebrates are pol-miR-101a, pol-miR-124-3p_R-2, pol-miR-140-3p_R + 1, pol-miR-140-5p, pol-miR-153-5p, pol-miR-153a, pol-miR-19a-5p_L-1R + 1, pol-miR-190a, pol-miR-199a-5p_R + 1, and pol-miR-455a_R-1. For example, pol-miR-19a-5p_L-1R + 1 exhibits homology with the miRNAs of age-mir-19a (spider monkey), tgu-mir-19a (zebra finch), hsa-miR-19a-5p (human), gga-miR-19a-5p (chicken), mml-miR-19a-5p (rhesus monkey), and mdo-miR-19a-5p (gray short-tailed opossum) (Additional file 2). In general, most of the homologues of flounder miRNAs were found in zebrafish.

### Identification of viral miRNAs

**Figure 2:**
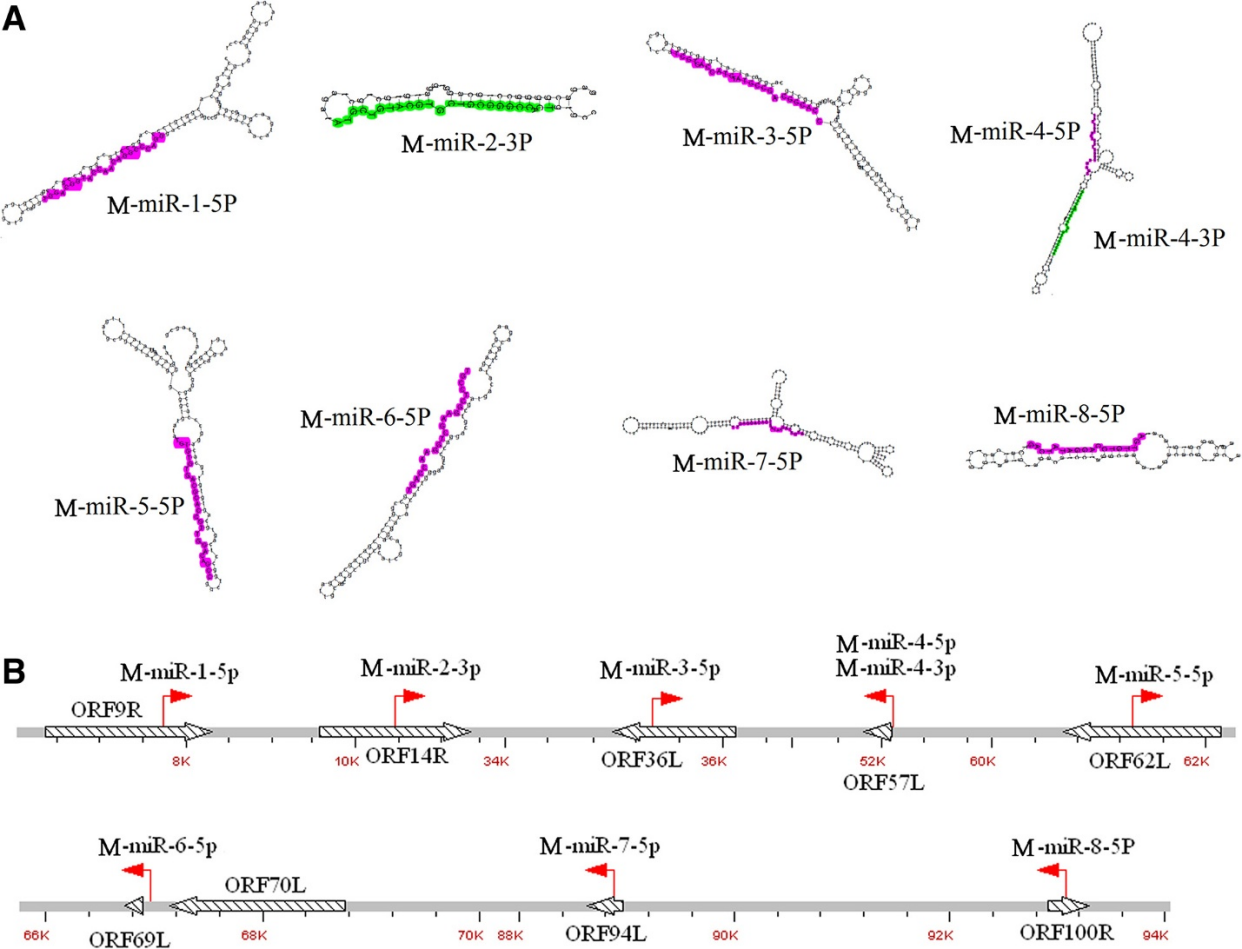
**The location and structure of novel megalocytivirus miRNAs. (A)** The candidate viral miRNAs within precursor miRNAs. Folding calculations were determined with Mfold algorithm. In each hairpin structure, pink indicates mature miRNAs located at the 5’-arm (5p), and green indicates mature miRNAs located at the3’-arm (3p). **(B)** Schematic diagrams of the genomic locations of the miRNAs. Hollow and striped arrows denote the relative size, location, and orientation of viral ORFs; small red arrows indicate the transcriptional orientation and genomic position of miRNAs.

Of the 50,746,504 mappable RNAs obtained above, 292 reads perfectly match the RBIV-C1 genome sequence. When these reads were aligned to known viral miRNAs deposited at miRBase 20.0, no homologues were found. The reads plus the extended sequences in RBIV-C1 genome were scanned to identify regions with hairpin-forming potential. Based on this potential and other characteristics defined in the principles described in Methods (section “Analysis of sequencing reads”), nine reads were identified as candidates of novel viral miRNAs (Additional file 1). These miRNAs distribute throughout the RBIV-C1 genome and are derived from eight imperfect fold-back structure precursors (Figure 2). Seven of these miRNAs are located at the 5’-arm (5p) or the 3’-arm (3p) of pre-miRNAs, and two, i.e., M-miR-4-5p and M-miR-4-3p, are derived from the same pre-miRNA (Figure 2A). Six miRNAs are located in coding regions, one (M-miR-6-5p) is between two adjacent coding regions, and two (M-miR-4-5p and M-miR-4-3p) overlap the terminus of the same coding region. Furthermore, of the six miRNAs located in coding regions, three are in the same transcription orientations as the corresponding coding regions, while the other three are transcribed from the opposite directions to the coding regions (Figure 2B).

### Expression patterns of the novel viral miRNAs during the course of infection

**Figure 3:**
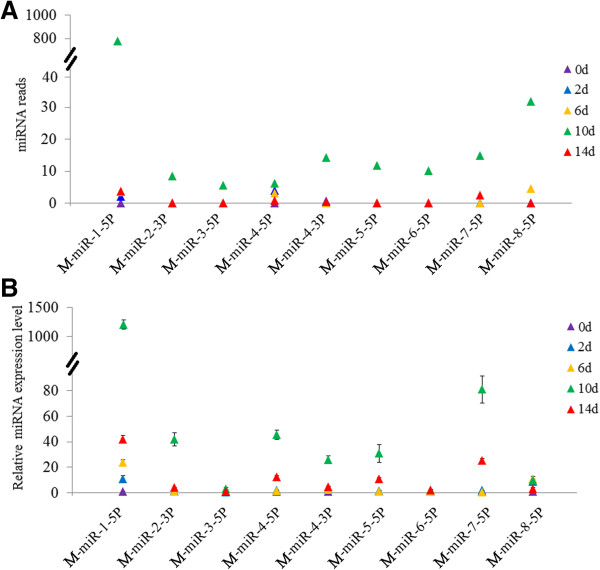
**Expression of the nine novel megalocytivirus miRNAs during the course of infection.** The expression levels of the viral miRNAs at different days (d) of infection were determined with high-throughput sequencing **(A)** and stem-loop relative quantitative RT-PCR **(B)**. In panel **B**, values are shown as means ± SEM (N =3). N, the number of times the experiment was performed.

To examine the expression levels of the nine novel viral miRNAs at different infection time points, the amounts of the miRNAs were normalized with Bonferroni correction. The results showed that all nine miRNAs increased as infection progressed until 10 d pi and then dropped at 14 d pi (Figure 3A). Only one miRNA, M-miR-1-5P, was detected at both early and late infection stages. To validate the authenticity of these expression patterns, stem-loop RT-PCR was performed to determine the expression levels of the nine miRNAs at 0 d pi, 2 d pi, 6 d pi, 10 d pi, and 14 d pi. The results showed that the expression profiles of all nine miRNAs were largely similar to those determined by high-throughput sequencing (Figure 3B).

### Differentially expressed host miRNAs induced by viral infection

**Figure 4:**
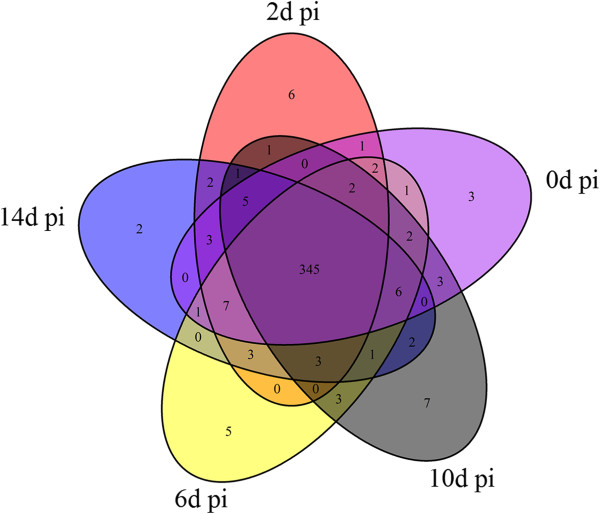
**Venn diagram of flounder miRNAs expressed at different days (d) post-viral infection (pi).** The numbers inside the diagram indicate the numbers of miRNAs.

**Figure 5:**
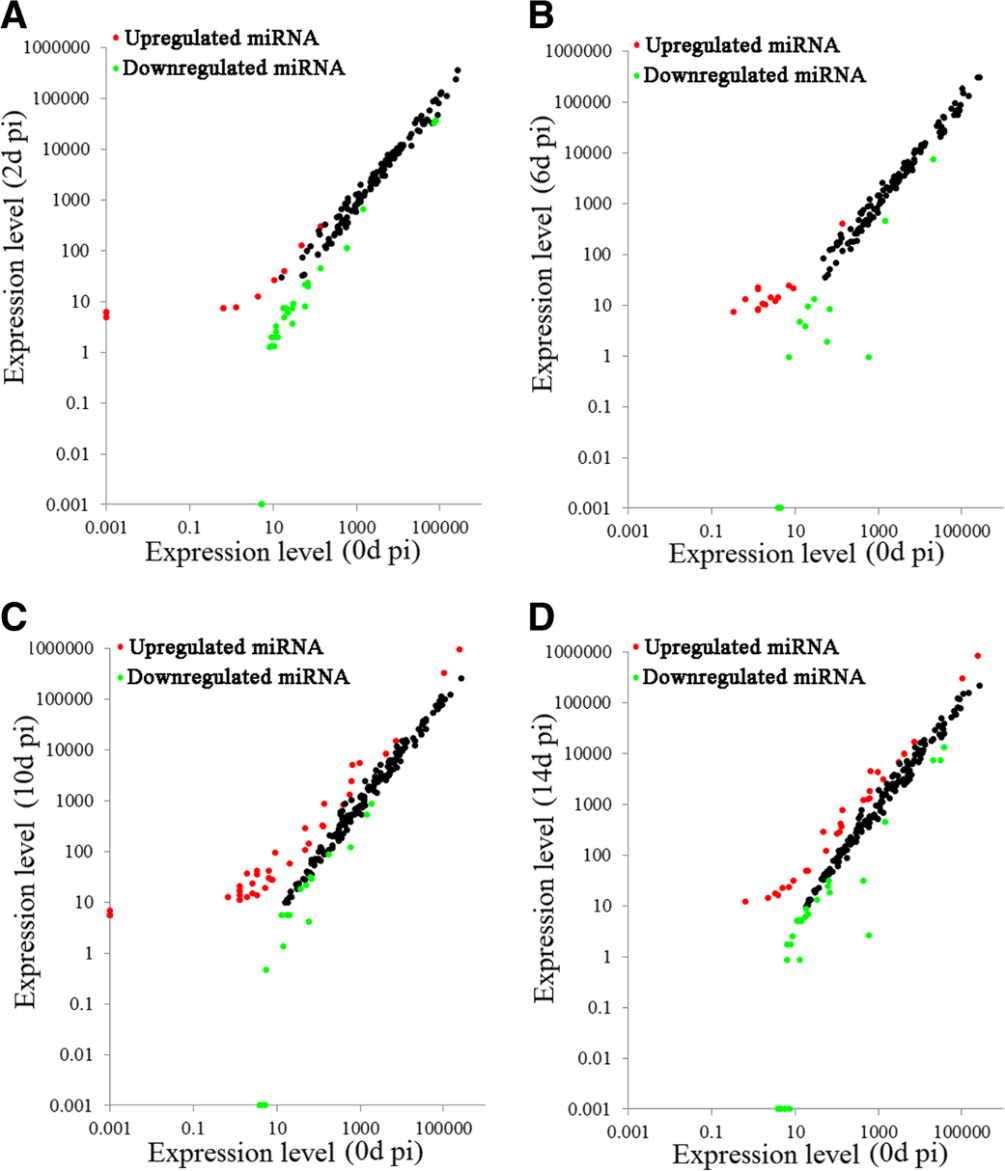
**Effect of megalocytivirus infection on the expression of flounder miRNAs.** Scatter plot of the expression levels of flounder miRNAs at 2 d **(A)**, 6 d **(B)**, 10 d **(C)**, and 14 d **(D)** post-viral infection (pi) in comparison with that at 0 d pi. Red and green spots represent miRNAs that are significantly (*P* < 0.01) upregulated and downregulated respectively.

**Figure 6:**
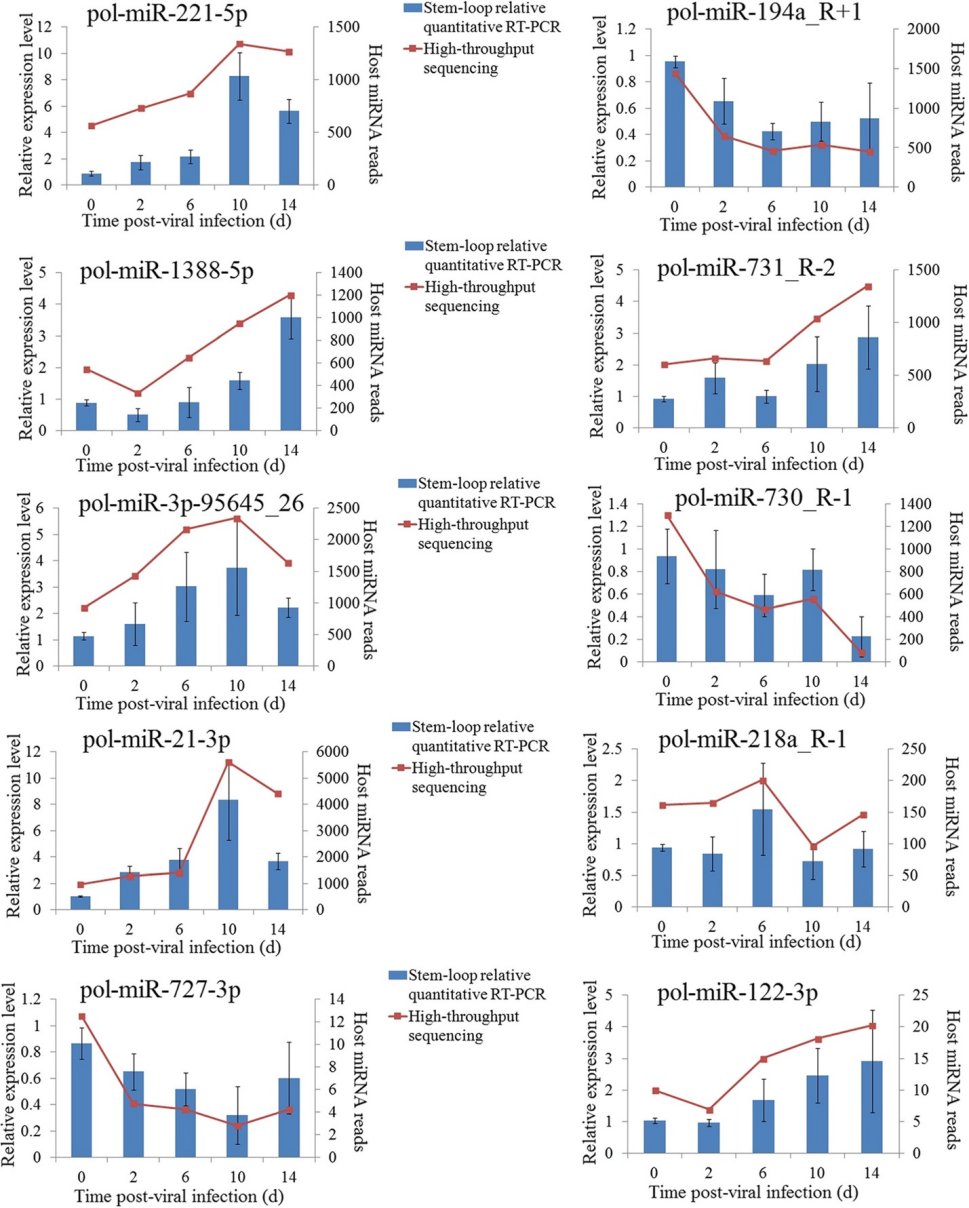
**Expression of ten flounder miRNAs during infection.** The expression levels of ten flounder miRNAs at different days (d) of infection were determined with stem-loop relative quantitative RT-PCR. Values are shown as means ± SEM (N =3). N, the number of times the experiment was performed.

**Figure 7:**
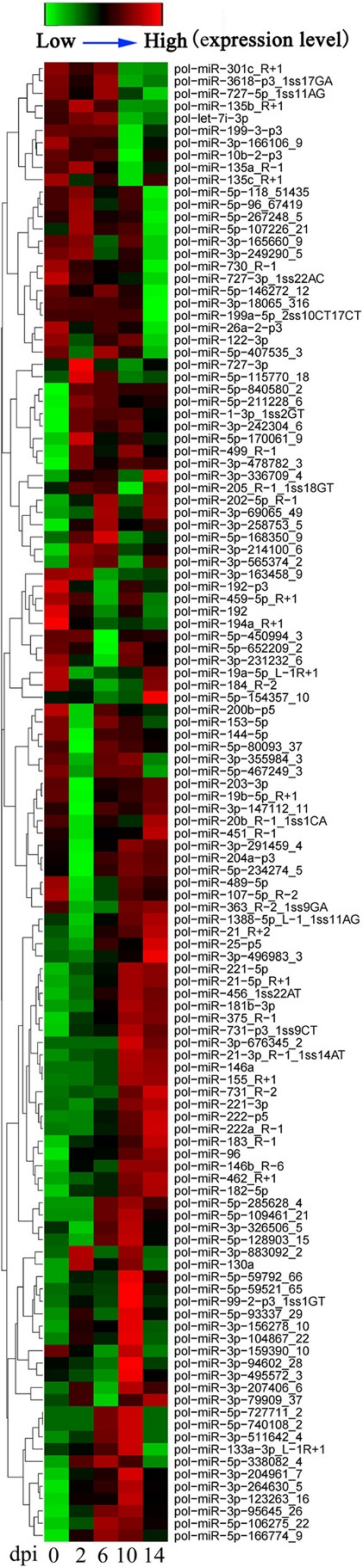
**Clustering of the expression patterns of 121 host miRNAs expressed differentially during viral infection.** The expression levels of the 121 miRNAs at 0 d to 14 d post-infection (dpi) are shown in different colors. Each horizontal color bar represents one miRNA, with the name of the miRNA indicated on the right of the bar.

To identify host miRNAs involved in viral infection, the expression profiles of the 381 flounder miRNAs were examined at 0 d pi, 2 d pi, 6 d pi, 10 d pi, and 14 d pi, and the amounts of miRNA were normalized with Bonferroni correction. The results showed that 345 miRNAs expressed at all examined time points, while three, six, five, seven, and two miRNAs expressed specifically at 0 d pi, 2 d pi, 6 d pi, 10 d pi, and 14 d pi respectively (Figure 4, Additional file 3). Compared to the expression levels at 0 d pi, the expression levels of 121 miRNAs at 2 d pi to 14 d pi were significantly (*P* < 0.01) changed. Specifically, 11 miRNAs (including pol-miR-430a-18-p5_1ss2GA, pol-miR-130a, and pol-miR-499_R-1) were significantly upregulated at 2 d pi, 30 miRNAs (including dre-miR-184_R-2, pol-miR-3p-214100_6, pol-miR-19b-5p_R + 1, pol-miR-459-5p_R + 1, pol-miR-194a_R + 1, and pol-miR-203-3p) were significantly downregulated at 2 d pi, 15 miRNAs (including pol-miR-3p-258753_5, pol-miR-202-5p_R-1, pol-miR-5p-166774_9, and pol-miR-5p-106275_22) were significantly upregulated at 6 d pi, 14 miRNAs (including, pol-miR-459-5p_R + 1, pol-miR-735-p3_1ss13CG, pol-miR-100-1-p3_1ss17TC, pol-miR-5p-450994_3, and pol-miR-5p-652209_2) were significantly downregulated at 6 d pi, 41 miRNAs (including pol-miR-3p-676345_2, pol-miR-3p-242304_6, pol-miR-5p-740108_2, and pol-miR-5p-285628_4) were significantly upregulated at 10 d pi, 19 miRNAs (including pol-miR-726_R + 1, pol-mir-734-p5_1ss2GT, pol-mir-100-1-p3_1ss17TC, and pol-mir-10b-2-p3) were significantly downregulated at 10 d pi, 28 miRNAs (including pol-miR-202-5p_R-1, pol-miR-155_R + 1, pol-miR-3p-496983_3, pol-miR-146b_R-6, and pol-miR-375_R-1) were significantly upregulated at 14 d pi, and 29 miRNAs (including pol-mir-100-1-p3_1ss17TC, pol-miR-199a-5p_2ss10CT17CT, pol-miR-5p-467249_3, pol-miR-146, pol-miR-221-5p, pol-miR-221-3p, and pol-miR-3p-192896_7) were significantly downregulated at 14 d pi (Figure 5A). To validate the expression patterns of the miRNAs, stem-loop RT-PCR was conducted to examine the mRNA levels of ten randomly selected miRNAs. The results showed that the expression profiles of all ten miRNAs were essentially similar to those determined by high-throughput sequencing (Figure 6). For all differentially expressed miRNAs, in order to observe their expressions along with the infection process, a heat map was drawn, and clustering analysis was conducted based on similar expression patterns (Figure 7). The results showed that all differentially expressed miRNAs were grouped together by *k*-means clustering. As the infection progressed, the expressions of the miRNAs exhibited dynamic changes and formed various patterns, including sustained upregulation/downregulation during the entire infection period, upregulation/downregulation followed by downregulation/upregulation, and diphasic expression patterns.

### Prediction of the target genes of host miRNAs

Potential targets of the 121 differentially expressed host miRNAs were predicted using Targetscan 6.2 and miRanda algorithms. The results showed that for 107 miRNAs, 800 putative target sites in 243 flounder genes were predicted (Additional file 4), while for the remaining 14 differentially expressed miRNAs, no target sites in flounder were found. The 243 putative target genes cover a wide range of functions, notably those related to immunity (Additional file 5: Table S1). Immune relevant genes include interleukin (IL)-8 targeted by pol-miR-3p-511642_4, interferon-induced Mx protein (Mx) targeted by pol-miR-199-3-p3 and pol-miR-459-5p_R + 1, T cell receptor (TCR) targeted by pol-miR-192-p3 and pol-miR-3p-242304_6, interferon regulatory factor (IRF) 7 targeted by pol-miR-194a_R + 1 and pol-miR-301c_R + 1, C-type lectin targeted by pol-miR-153-5p, pol-miR-200b-p5 and pol-miR-20b_R-1_1ss1CA, complement component 3 (C3) targeted by pol-miR-203-3p, p65 NF-kB targeted by pol-miR-221-3p and pol-miR-222a_R-1, toll-like receptor (TLR) 3 targeted by pol-miR-129-3p, pol-miR-221-5p, pol-miR-221-3p, and pol-miR-200b-p5, myeloid differentiation factor 88 (Myd88) targeted by pol-miR-146a, cathepsin B targeted by pol-miR-203-3p, type 1 insulin-like growth factor receptor fIGF-IR-2 targeted by pol-miR-194a_R + 1 and pol-miR-459-5p_R + 1, and transcription factor PU.1 targeted by pol-miR-459-5p_R + 1.

### Enrichment analysis of the putative target genes of host miRNAs

**Figure 8:**
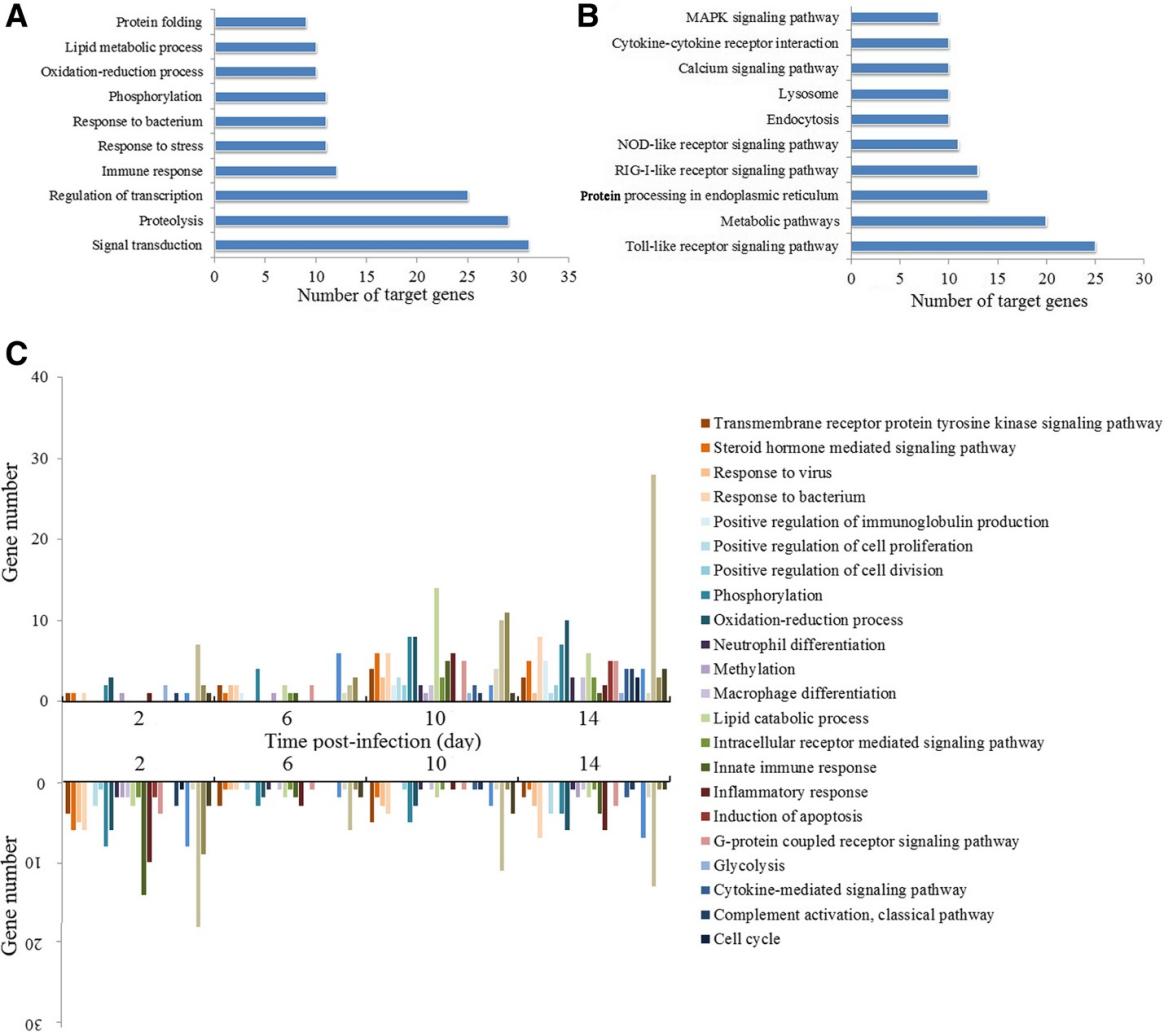
**Pathways predicted to be affected by differentially expressed flounder miRNAs during viral infection. (A)** Top ten enriched GO terms of biological process for the target genes of flounder miRNAs. **(B)** Top ten abundant pathways from KEGG pathway analysis of the target genes of flounder miRNAs. **(C)** The enriched GO terms of biological process for the target genes of flounder miRNAs at different time points of viral infection.

To get an overview of the pathways in which host miRNAs were involved, the putative target genes of the differentially expressed host miRNAs were subjected to GO analysis and KEGG pathway analysis. GO enrichment analysis based on biological process showed that the 243 predicted target genes of flounder were clustered into 296 GO terms. The top ten enriched GO terms are associated with signal transduction, proteolysis, transcription regulation, immune response, stress and infection response, phosphorylation, oxidation-reduction process, lipid metabolic process, and protein folding (Figure 8A). To examine the effect of the miRNAs in more detail, another GO analysis was conducted, in which the 121 differentially expressed host miRNAs were grouped into upregulated and downregulated categories at each infection time point, and then GO analysis was performed on the target genes of the miRNAs in each category. The results revealed an interesting trend, i.e., the numbers of target genes in all pathways changed at different time points (Figure 8C). For example, at 2 d pi, the genes belonging to the GO terms of proteolysis, apoptotic process, innate immune response (in particular inflammatory response), antigen processing and presentation, and phosphorylation were preferably targeted by downregulated miRNAs; at 6d pi, relatively few target genes were enriched into GO terms; at 10 d pi, fourteen, ten, and eleven genes targeted by downregulated miRNAs were enriched into the processes of lipid catabolism, apoptosis, and antigen processing/presentation respectively; at 14 d pi, genes involved in apoptotic process were the highest in number among the genes targeted by upregulated miRNAs.

Similar to GO analysis, KEGG pathway analysis showed that the putative target genes predicted in flounder were grouped into 64 pathways. The top ten enriched pathways were involved in TLR signaling, metabolism, protein processing in endoplasmic reticulum, RIG-I-like receptor signaling, NOD-like receptor (NOD-RC) signaling, endocytosis, lysosome, calcium signaling, cytokine-cytokine receptor interaction, and MAPK signaling (Figure 8B).

### Prediction and enrichment analysis of the putative target genes of viral miRNAs

**Figure 9:**
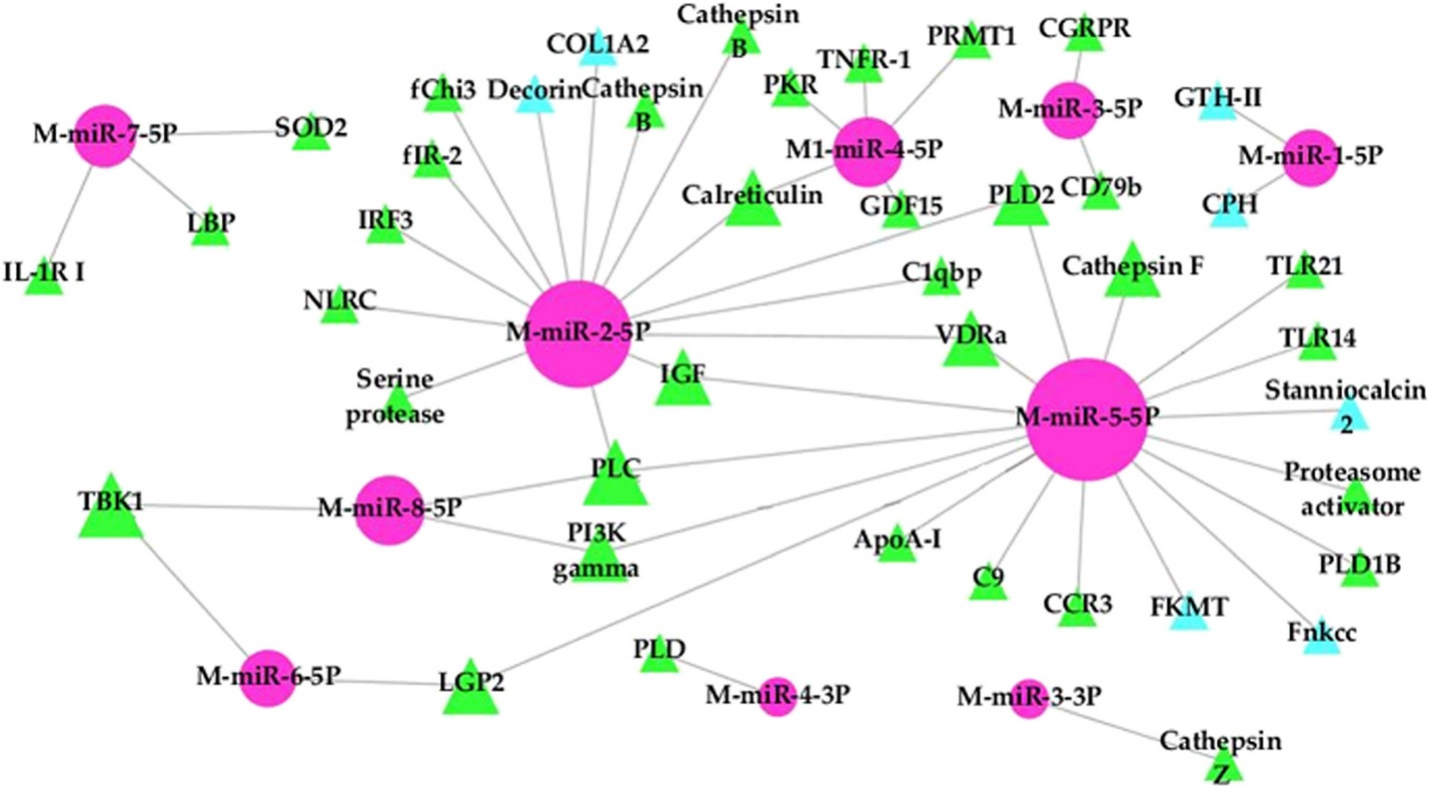
**A network of putative interactions between megalocytivirus miRNAs and the target genes in flounder.** Pink indicates viral miRNAs; green indicates target genes known to be associated with immune response or apoptosis; light blue indicates target genes that have no known association with immune response or apoptosis process. Abbreviations of the genes are detailed in the text.

For the nine novel viral miRNAs, target prediction identified 56 putative target sites in flounder, which are distributed among 43 genes. GO analysis showed that most of the predicted target genes of viral miRNAs are related to signal transduction and immune response. The immune relevant genes include TLR14, IRF3, NOD-RC, tumor necrosis factor receptor-1 (TNFR-1), voltage-dependent anion channel (VDAC), and complement component 9 (C9), which are targeted by M-miR-5-5P, M-miR-2-5P, M-miR-2-5P, M-miR-4-5P, M-miR-7-5P, and M-miR-5-5P respectively (Figure 9). These putative target genes were all clustered into the innate immunity GO term. M-miR-4-5P was also predicted to target double-stranded (ds) RNA-dependent kinase (PKR), growth differentiation factor-15 (GDF-15), and tumor necrosis factor alpha (TNF-α). TNF-α was grouped into the GO terms of apoptotic process and innate immunity. For the remaining miRNAs, M-miR-3-5P was predicted to target immunoglobulin β (CD79B), which was assigned to adaptive immunity GO term; M-miR-2-3P and M-miR-5-5P target cathepsin B, F, and Z, which were classified into lysosome GO term. In addition, of the 43 flounder genes targeted by viral miRNAs, 39 genes were also targeted by host miRNAs; however, no identical target sites in these genes were found for host and viral miRNAs.

## Discussion

In this study, we identified and investigated the expression patterns of both viral and host miRNAs in a teleost fish, Japanese flounder, infected with megalocytivirus at different time points. We detected 381 host miRNAs, of which 130 were discovered for the first time. These novel miRNAs add to the known miRNAs pools of fish. Previous studies showed that viral infection can profoundly influence the expression of host miRNAs [36–41]. In humans, distinct miRNA expression profiles were observed after HIV infection [42, 43]. Likewise, avian influenza virus infection changed the expression of a large amount of miRNAs in the lung and trachea of chicken [21]. Similar studies in lower vertebrates, including fish, have not been reported before this study. In this study, we found that 121 host miRNAs displayed significantly different expressions during viral infection, suggesting that megalocytivirus altered, on a large scale, the regulation of miRNA expression in flounder. These results indicate the possibility that, as observed in higher vertebrates, miRNAs likely participate in host-virus interaction in teleost.

Detailed analysis of the expression profiles of flounder miRNAs on a timescale of 14 days revealed that dynamic changes were associated with the course of megalocytivirus infection, and that the expression levels of individual miRNAs varied at different time points. These observations support the hypothesis that different miRNAs may have different roles, which are played out at different infection stages. It is possible that the differentially expressed host miRNAs may act on the host itself, such as regulating gene expression in flounder, or on virus, such as modifying the expression of the genes of megalocytivirus. For regulation of host genes, two possibilities exist, one is that the host miRNAs actively exert their functions in a natural manner that promotes viral clearance, and the other is that the host miRNAs are subverted by virus to serve as mediators of viral infection. Evidences of both situations have been demonstrated in higher vertebrates, e.g., the human miRNAs miR-146a and miR-29b are induced during EBV infection, which leads to downregulation of interferon-responsive genes [40–45].

For the 121 differentially expressed host miRNAs identified in our study, 243 putative target genes were predicted in flounder. GO and KEGG pathway analysis showed that the predicted target genes are involved in diverse biological processes ranging from fundamental cellular operations to stress response, suggesting that miRNAs may have a global impact on the host system. It is of note that the majority of the differentially expressed host miRNAs were downregulated at early infection time (2d pi). Since miRNAs function to suppress gene expression [46], this phenomenon implies that the genes associated with many crucial cellular pathways are probably upregulated in response to megalocytivirus infection. Of these downregulated miRNAs, pol-miR-19a-5p_L-1R + 1 is a homologue of the human miRNA hsa-miR-19a-5p, which regulates a wide range of pathways in many diseases through targeting PTEN (phosphatase and tensin homolog deleted on chromosome ten) [47], cyclin D1 [48], and TNF-α [49]. Of the putative target genes of the downregulated miRNAs, the transcription factor PU.1 is known to be required for Flt3 cytokine receptor expression and for the development of IL-9-producing T cells and dendritic cells [50, 51], fIGF-IR-2 has been reported to protect cells from apoptosis [52, 53], whereas cathepsin B is known to contribute to TNF-α–mediated apoptosis [54, 55]. Apoptosis is generally considered an innate response of the host to counteract viral infection [56]. Consequently, for virus in infection, it is necessary to inhibit the apoptotic process so to prevent premature destruction of the host cells and enable viral production at the later infection stage [57]. The observation in our study of both pro- and anti-apoptosis targets of miRNAs suggests that some of the flounder miRNAs operate to promote host defense, while others may serve for virus infection.

Another striking feature observed with the differentially expressed flounder miRNAs in our study is that at 10 d pi and 14 d pi, the numbers of upregulated miRNAs dramatically increased compared to those at 2 d pi to 6 d pi. Similar observations have been made with human epithelial A549 cells infected with H1N1 influenza virus [58]. The upregulated miRNAs in our study included pol-miR-146, whose homologues in mammals are known to function in a negative feedback pathway of TLR and cytokine signaling [23, 59] and to act as negative regulators of interferon signaling by targeting IRF5 and STAT-1 transcription factors [60]. Thus, in flounder, enhanced expression of pol-miR-146 may remove the negative holds on cytokine and interferon signaling, whereby augmenting antiviral defense. Two other upregulated miRNAs, pol-miR-221-5p and pol-miR-221-3p, were predicted to target flounder phospholipase C. In humans, phospholipase C is a key enzyme that hydrolyzes phosphatidylinositol 4,5-bisphosphate to trigger calcium release from intracellular stores, which facilitates virus entry into host cells [61, 62]. Given these previous findings, the upregulation of pol-miR-221-5p and pol-miR-221-3p may, through inhibiting phospholipase C expression, represent a novel way to prevent viral spread in flounder.

Aside from host-encoded miRNAs, our study also discovered 9 novel viral miRNAs encoded by the RBIV-C1 genome. Most of the viral miRNAs are located within coding regions, which suggests the possibility that megalocytivirus may, like other viruses [63], use its coding capacity to the maximum. As observed with herpes simplex virus [64, 65], two megalocytivirus miRNAs were derived from the 5’- and 3’-arm of the same precursor miRNA. However, the reads of these two miRNAs are different, which is probably the result of difference in processing, as a recent report showed that the processing of 3p- and 5p-derived mature miRNAs depends on the RNase IIIB and RNase IIIA domains of Dicer respectively [66]. Two of the identified miRNAs, i.e. M-mir-7-5p and M-mir-4-5p, are not typical form of miRNA precursors, nevertheless they were considered miRNAs based on the observations that (i) the corresponding mature miRNAs were detected and identified with stem loop RT-PCR, and (ii) many of the predicted target genes of these miRNAs are grouped into innate immune response GO term and/or apoptotic process GO term, both which play crucial roles in antiviral defense or viral immune evasion. It is interesting that none of the megalocytivirus miRNAs shares homology with known viral miRNAs, which may be due to the fact that to date very little study on iridovirus miRNAs has been documented, and, except for a report on the miRNA of Singapore grouper iridovirus, an iridovirus of the genus *Ranavirus* [67], no miRNAs from other genera, including *Megalocytivirus*, have been identified. Another reason could be that in order to adapt rapidly to specific host environments, viral miRNAs are capable of higher rate of mutation and faster evolution compared to cellular miRNAs [38, 63]. It is noteworthy that, unlike previous studies such as that by Skalsky et al., in which a miRNA of Kaposi's sarcoma-associated herpesvirus was found to share 100% seed sequence homology with a human miRNA [68], the viral miRNAs identified in our study failed to match any known host miRNAs. This observation suggests that megalocytivirus miRNAs may not be able to regulate the expression of host mRNAs through evolutionarily conserved target sites as reported for the viral miRNAs with orthologs in cellular miRNAs [38].

Target prediction and GO analysis showed that megalocytivirus miRNAs have putative targets in the 3’-UTRs of a number of immune genes. Of these genes, TLR14 is proposed to be a functional substitute of mammalian TLR6 and TLR10 and participate in immune response against a wide variety of pathogens in finfish [69], IRF3 is essential for inducible expression of Type I IFN genes [70, 71], and Igβ is required for B cell development and maintenance [72, 73]. In addition to immune genes, genes involved in host apoptosis were also predicted as targets of megalocytivirus miRNAs. Of these genes, VDAC is a key functional target of the Bcl-2 family of proteins and involved in regulating mitochondrial membrane permeability during apoptosis [74], GDF-15 is a transcriptional target of p53 family members, and overexpression of GDF-15 promotes apoptosis in human breast and colorectal cell lines [75, 76]. Other putative targets of megalocytivirus miRNAs include PKR, which is known to induce apoptosis by activation of the FADD/caspase 8 pathway and prevent virus replication by inhibiting translation of viral mRNAs through phosphorylation of eIF2α [77, 78]. Taken together, these observations lend support to the notion that miRNAs facilitate megalocytivirus infection by manipulating host immune response and prolonging survival of infected host cells.

## Conclusions

In conclusion, we in this study identified 9 novel megalocytivirus miRNAs and 381 flounder miRNAs, the latter including 130 novel miRNAs and 121 differently expressed miRNAs. The 9 viral miRNAs and the 121 differently expressed host miRNAs exhibited dynamic changes in expression during the course of viral infection. The putative targets of host and viral miRNAs were grouped into a wide range of functional categories, in particular those associated with immune defense/evasion and signal transduction. These results suggest that in teleost, as in higher vertebrates, miRNAs likely have profound effects on both host immune defense and viral infection. In addition, the large amount of novel miRNAs as well as their putative target genes identified in this study will serve as a useful source for future researches to investigate, from a new angle, the mechanisms of both viral infection and host defense in teleost.

### Availability of supporting data

The data supporting the results of this article are included in the article and its additional files.

## Electronic supplementary material

Additional file 1: **Primers for stem-loop RT-PCR.** (XLS 22 KB)

Additional file 2: **The sequences and homologues of 381 flounder miRNAs.** (XLSX 29 KB)

Additional file 3: **Expression frequency of flounder miRNAs.** (XLSX 40 KB)

Additional file 4: **Putative miRNA targets in flounder.** (XLSX 31 KB)

Additional file 5: **Putative target genes of differentially expressed host miRNAs associated with immune response.** (DOCX 15 KB)

## Abbreviations

apoA-I: Apolipoprotein A-I
C1qbp: Complement component 1q subcomponent gamma polypeptide
C9: Complement component C9
CCR3: C-C chemokine receptor-3
CD79b: immunoglobulin β (CD79B)
cgrpr: Calcitonin gene-related peptide receptor
COL1A2: Type 1 collagen alpha2
CPH: Carboxypeptidase H
fChi3: chitinase3
fIR-2: IR-2 insulin receptor
FKMT: Fast skeletal muscle troponin
Fnkcc: Na-K-Cl cotransporter
GDF15: Growth differentiation factor 15
GTH-II: Gonadotropin beta subunit
IGF: Insulin-like growth factor I
IL-1R I: Interleukin-1 receptor type II
IRF3: Interferon regulatory factor 3 variant 1
LBP: Lipopolysaccharide-binding protein/bactericidal permeability-increasing protein
LGP2: ATP-dependent helicase LGP2
NLRC: NOD-like receptor C
PI3K gamma: Phosphoinositide 3-kinase gamma
PKR: dsRNA-dependent protein kinase
PLC: Phospholipase C
PLD: Phospholipase D
PLD1B: Phospholipase D1B
PLD2: Phospholipase D2
PRMT1: Arginine methyltransferase 1
SOD2: Mn-superoxide dismutase
TBK1: TANK binding kinase 1
TLR14: Toll like receptor 14
TLR21: Toll-like receptor 21
TNFR-1: Tumor necrosis factor receptor-1
VDRa: Vitamin D receptor a.
