# Supplementary material for: In-depth profiling and analysis of host and viral microRNAs in Japanese flounder (Paralichthys olivaceus) infected with megalocytivirus reveal involvement of microRNAs in host-virus interaction in teleost fish
Source: BMC Genomics. 2014 Oct 8;15(1):878. doi: 10.1186/1471-2164-15-878 (PMC4200114; doi:10.1186/1471-2164-15-878)
Supplement: Supplementary file 5 — Additional file 5: Putative target genes of differentially expressed host miRNAs associated with immune response. (DOCX 15 KB) [file 12864_2014_6551_MOESM5_ESM.docx]

**Table S1**. Target genes of differentially expressed flounder miRNAs associated with immune response

| miRNA | Target gene |
| --- | --- |
| pol-miR-203-3p, pol-miR-153-5p, pol-miR-375_R-1, pol-miR-3p-336709_4, pol-miR-5p-146272_12 | B cell accessory protein (CD79b) |
| pol-miR-204a-p3, pol-miR-211-3p_1ss18GA, pol-miR-726_R+1 | Calmodulin |
| pol-miR-199a-5p_R+1, pol-miR-21-3p_R-1_1ss14AT, pol-miR-3p-147112_11, pol-miR-5p-59521_65 | C-C chemokine receptor 9 (CCR9) |
| pol-miR-222a_R-1, pol-miR-3p-355984_3 | C-C chemokine receptor-3 (CCR3) |
| pol-miR-130a, pol-miR-301c_R+1, pol-miR-3p-147112_11, pol-miR-3p-511642_4, pol-miR-489-5p, pol-miR-5p-170061_9, pol-miR-5p-407535_3, pol-miR-731_R-2 | CD40 |
| pol-miR-107-5p_R-2, pol-miR-1388-5p_L-1_1ss11AG, pol-miR-181b-3p, pol-miR-21-3p_R-1_1ss14AT, pol-miR-3p-565374_2, pol-miR-5p-168350_9 | CD8 alpha chain |
| pol-miR-203-3p | Complement component C3 |
| pol-miR-192-3p_R-1, pol-miR-192-p3, pol-miR-21-3p_R-1_1ss14AT, pol-miR-3p-159390_10, pol-miR-5p-80093_37 | Complement component C9 |
| pol-miR-153-5p, pol-miR-200b-p5, pol-miR-20b_R-1_1ss1CA, pol-miR-5p-146272_12, pol-miR-5p-710312_2 | C-type lectin |
| pol-miR-153-5p, pol-miR-21-5p_R+1, pol-miR-3p-123263_16, pol-miR-3p-163458_9, pol-miR-5p-128903_15, pol-miR-5p-211228_6, pol-miR-5p-372186_3, pol-miR-727-5p_1ss11AG, pol-miR-727-5p_R+2_1ss11AG, pol-miR-731_R-2 | Granulocyte Colony-Stimulating Factor(G-CSF) |
| pol-miR-107-5p_R-2, pol-miR-3p-104867_22, pol-miR-3p-147112_11, pol-miR-459-5p_R+1, pol-miR-5p-727711_2, pol-miR-727-3p, pol-miR-727-3p_1ss22AC | IL-1b |
| pol-miR-146a, pol-miR-146b_R-6, pol-miR-21-3p_R-1_1ss14AT, pol-miR-5p-338082_4, pol-miR-5p-520972_3 | Insulin receptor (fIR-2) |
| pol-miR-153-5p, pol-miR-155_R+1, pol-miR-199-3-p3, pol-miR-3p-159390_10, pol-miR-3p-355984_3, pol-miR-3p-478782_3, pol-miR-3p-511642_4, pol-miR-460-3p, pol-miR-5p-407535_3, pol-miR-5p-520972_3, pol-miR-5p-740108_2, pol-miR-3p-355984_3 | Insulin-like growth factor I (IGF-1) |
| pol-miR-3p-147112_11 | Interferon regulatory factor 3 variant 1 (IRF3) |
| pol-miR-194a_R+1, pol-miR-301c_R+1, pol-miR-5p-146272_12, pol-miR-5p-407535_3, pol-miR-5p-727711_2, pol-miR-727-3p, pol-miR-727-3p_1ss22AC, pol-miR-727-3p_1ss22AC, pol-miR-731_R-2 | Interferon regulatory factor 7 (IRF7) |
| pol-miR-3p-511642_4 | Interleukine-8 (IL-8) |
| pol-miR-3p-511642_4, pol-miR-3p-511642_4, pol-miR-3p-565374_2, pol-miR-5p-109461_21, pol-miR-5p-521683_3 | MHC class II |
| pol-miR-199-3-p3, pol-miR-459-5p_R+1, pol-miR-5p-267248_5, pol-miR-5p-407535_3 | Mx |
| pol-miR-146a, pol-miR-3p-478782_3, pol-miR-3p-565374_2, pol-miR-3p-676345_2, pol-miR-5p-727711_2 | Myeloid differentiaton factor 88 (MyD88) |
| pol-miR-144-5p, pol-miR-194a_R+1 | NOD-like receptor C (NLRC) |
| pol-miR-205_R-1_1ss18GT, pol-miR-221-3p, pol-miR-222a_R-1, pol-miR-3p-231232_6, pol-miR-3p-242304_6, pol-miR-459-5p_R+1 | p65 NF-kB |
| pol-miR-192-3p_R-1, pol-miR-192-p3, pol-miR-3p-242304_6, pol-miR-3p-676345_2, pol-miR-5p-234274_5, pol-miR-5p-407535_3, pol-miR-5p-450994_3, pol-miR-5p-59521_65, pol-miR-5p-710312_2, pol-miR-3p-147112_11, pol-miR-3p-165660_9 | T cell receptor (TCR) |
| pol-miR-3618-p3_1ss17GA, pol-miR-375_R-1, pol-miR-3p-163458_9 | T-cell surface glycoprotein CD4 |
| pol-miR-3p-676345_2, pol-miR-5p-115770_18, pol-miR-727-5p_1ss11AG, pol-miR-727-5p_R+2_1ss11AG | T-cell surface glycoprotein CD8 beta (CD8 beta) |
| pol-miR-192-3p_R-1, pol-miR-192-p3, pol-miR-203-3p, pol-miR-3p-123263_16, pol-miR-5p-146272_12, pol-miR-5p-267248_5, pol-miR-5p-407535_3 | Toll like receptor 14 (TLR 14) |
| pol-miR-129-3p, pol-miR-153-5p, pol-miR-200b-p5, pol-miR-3p-242304_6, pol-miR-3p-264630_5, pol-miR-3p-326506_5, pol-miR-5p-840580_2, pol-miR-726_R+1, pol-miR-728-p5 | Toll-like receptor 3 (TLR3) |
| pol-miR-155_R+1, pol-miR-222-p5, pol-miR-459-5p_R+1 | Transcription factor PU.1 |
| pol-let-7a-2-3p_1ss8GA, pol-miR-130a, pol-miR-192, pol-miR-21-3p_R-1_1ss14AT, pol-miR-5p-727711_2, pol-miR-727-3p, pol-miR-727-3p_1ss22AC, pol-miR-731_R-2, pol-miR-194a_R+1, pol-miR-363_R-2_1ss9GA, pol-miR-3p-147112_11, pol-miR-5p-93337_29 | Tumor necrosis factor receptor-1 (TNFR-1) |
